# Efficacy and Tolerability of Intranasal Midazolam Administration for Antiseizure Treatment in Adults: A Systematic Review

**DOI:** 10.1007/s12028-024-01971-x

**Published:** 2024-04-05

**Authors:** Tolga D. Dittrich, Dominik Vock, Urs Fisch, Lisa Hert, Sira M. Baumann, Paulina S.C. Kliem, Stephan Rüegg, Stephan Marsch, Gian Marco De Marchis, Raoul Sutter

**Affiliations:** 1 Department of Neurology and Stroke Center, University Hospital Basel and University of Basel, Basel, Switzerland; 2 Department of Clinical Research, University of Basel, Basel, Switzerland; 3 Department of Neurology and Stroke Center, Cantonal Hospital St. Gallen, St. Gallen, Switzerland; 4 Intensive Care Unit, Department of Acute Medical Care, University Hospital Basel and University of Basel, Petersgraben 4, 4031 Basel, Switzerland; 5 Medical Faculty, University of Basel, Basel, Switzerland

**Keywords:** Midazolam, Intranasal, Seizure, Status epilepticus, Epilepsy

## Abstract

**Objective:**

The objective of this study was to assess the efficacy and tolerability of intranasal midazolam (in-MDZ) administration for antiseizure treatment in adults.

**Methods:**

Embase and Medline literature databases were searched. We included randomized trials and cohort studies (excluding case series) of adult patients (≥ 18 years of age) examining in-MDZ administration for epilepsy, epileptic seizures, or status epilepticus published in English between 1985 and 2022. Studies were screened for eligibility based on predefined criteria. The primary outcome was the efficacy of in-MDZ administration, and the secondary outcome was its tolerability. Extracted data included study design, patient characteristics, intervention details, and outcomes. Risk of bias was assessed using the Cochrane Risk of Bias Tool.

**Results:**

A total of 12 studies with 929 individuals treated with in-MDZ were included. Most studies were retrospective, with their number increasing over time. Administered in-MDZ doses ranged from 2.5 to 20 mg per single dose. The mean proportion of successful seizure termination after first in-MDZ administration was 72.7% (standard deviation [SD] 18%), and the proportion of seizure recurrence or persistent seizures ranged from 61 to 75%. Most frequent adverse reactions to in-MDZ were dizziness (mean 23.5% [SD 38.6%]), confusion (one study; 17.4%), local irritation (mean 16.6% [SD 9.6%]), and sedation (mean 12.7% [SD 9.7%]).

**Conclusions:**

Administration of in-MDZ seems promising for the treatment of prolonged epileptic seizures and seizure clusters in adults. Limited evidence suggests that intranasal administration is safe. Further research is warranted because of the heterogeneity of cohorts, the variation in dosages, and the lack of uniformity in defining successful seizure termination.

**Supplementary Information:**

The online version contains supplementary material available at 10.1007/s12028-024-01971-x.

## Introduction

Rapid antiseizure treatment is crucial for persistent, prolonged, or recurrent epileptic seizures. The majority of epileptic seizures spontaneously cease within a few minutes [1]. However, cases that involve prolonged convulsive seizure activity (more than 5 min) or clusters of convulsive seizures without complete remission are defined as convulsive status epilepticus. If left untreated, status epilepticus can result in life-threatening systemic complications [2].

In those cases, rapid administration of antiseizure medications is of great importance but can be challenging in the acute care setting given the background of convulsions and the difficulty in obtaining rapid intravenous access. Benzodiazepines are the recommended first-line therapy for treating status epilepticus [3–5]. They exert their antiseizure effects by modulation of inhibitory GABA_A_ receptors, with each agent exhibiting different pharmacokinetic and pharmacodynamic properties [4, 6].

Midazolam administered intranasally may be an alternative when intravenous access is not available [4]. Intranasal absorption occurs mainly through the epithelium via olfactory and trigeminal nerve pathways into the central nervous system [7]. Intranasal application results in rapid peak plasma concentrations (mean at 14 min) and is associated with high bioavailability (mean 83%) [8]. These results are comparable to that after intramuscular administration (mean peak plasma concentration at 25 min; mean bioavailability 87%) [9]. Although clinicians appreciate the convenience of midazolam nasal spray, studies on intranasal midazolam (in-MDZ) administration have primarily focused on pediatric cohorts and its use for sedation purposes [10–13]. Recently, intramuscular administration of midazolam has gained increasing attention. In a study published after our screening period, a direct comparison with in-MDZ revealed that intramuscular administration was more frequently associated with severe hypotension, which may be a further advantage for in-MDZ administration (especially in out-of-hospital emergency settings without immediate monitoring options) [14]. Another recent retrospective study examined the real-world practice regarding the efficacy of midazolam at different doses and routes of administration [15]. It was shown that the midazolam dosage had an impact on clinical outcomes. It was concluded that intranasal administration might be less effective in terminating status epilepticus compared to intramuscular administration, although the observed differences may also be due to residual confounding associated with the retrospective design.

To date, the efficacy and tolerability of in-MDZ administration for treating epileptic seizures and status epilepticus in adults have not been systematically reviewed. We therefore sought to perform a systematic review regarding the use of in-MDZ administration in adults with epilepsy, epileptic seizures, or status epilepticus and compile information on its efficacy and safety from the literature.

## Methods

### Registration and Reporting

This systematic review was registered on the International Prospective Register for Systematic Reviews (PROSPERO) and followed the Preferred Reporting Items for Systematic Reviews and Meta-Analyses guidelines. The search and study selection were conducted after study registration on November 1, 2022 (PROSPERO study ID: CRD42022369040).

### Ethical Standards

This study was conducted in compliance with the ethical standards outlined in the Declaration of Helsinki and its amendments.

### Databases and Search Strategy

Two databases (Embase and Medline) were screened by one reviewer (TDD) using the Ovid interface. The search aimed to identify articles in English related to efficacy and tolerability aspects of in-MDZ application as antiseizure treatment in adults. The search period was from January 1, 1985, to October 1, 2022. A predefined search algorithm was used to identify eligible studies (details in Supplemental Text Sect. 1).

### Eligibility Criteria and Study Selection

We assessed the study eligibility based on the following criteria, all of which had to be met for a study to be included: (1) study on humans; (2) study that includes adult patients (i.e., ≥ 18 years); (3) study of in-MDZ application in the context of either epilepsy, epileptic seizure(s), or status epilepticus; (4) a randomized clinical trial or observational cohort study (excluding case series); (5) publication written in English language; and (6) study publication between January 1, 1985 and October 1, 2022.

Three reviewers (TDD, DV, and RS) screened the studies for eligibility and manually filtered the retrieved studies to exclude those not fulfilling the previously outlined eligibility criteria. The remaining collaborators reviewed their decisions. The final decision to include studies was made by majority consensus. An online literature management program was used for the screening and selection process (see Data extraction and synthesis).

### Data Extraction and Synthesis

Authors, year of publication, participating country(ies), study design, number of patients treated with in-MDZ, information on the underlying condition, applied midazolam dosage, comparators, and outcomes of interest (as outlined in the Outcomes of interest section) were extracted and archived by DV using the freely available SRDR + (Systematic Review Data Repository) online tool. Two reviewers (TDD and RS) subsequently reviewed and sorted the extracted data according to the study design. Disagreements were resolved through majority agreement. Corresponding authors were contacted and asked for unreported or missing data.

### Outcomes of Interest

The primary outcome was the efficacy of in-MDZ administration in terms of seizure termination (defined as suppressed clinical seizure activity) and recurrence (after first in-MDZ application with subsequent suppressed clinical seizure activity). The secondary outcome was the tolerability, reported side effects, or adverse events of in-MDZ administration. The latter was considered exploratory because we recorded all reported side effects in temporal relation to the in-MDZ administration and (if applicable) midazolam with different administration routes or other benzodiazepines.

### Risk of Bias Assessment

Risk of bias (ROB) assessment was conducted using the Cochrane Risk of Bias Tool for randomized trials and the Risk of Bias in Non-Randomized Studies of Interventions tool. Both tools are standardized and include a systematic assessment of the study design, implementation, and reporting of results.

### Evidence Rating

Studies comparing in-MDZ administration with a control intervention (another benzodiazepine[s], midazolam with another administration route, or placebo) were assigned an evidence rating using the Grading of Recommendations Assessment, Development, and Evaluation approach [16]. The secondary outcome, which was exploratory in nature, did not undergo rating.

### Statistics

Descriptive statistical analyses were performed to obtain relative frequencies (percentages and standard deviations [SDs]). For the certainty rating, risk ratios and absolute effects were calculated. STATA version 17.0 (StataCorp LLC, College Station, TX) was used for all analyses.

## Results

### Study Selection and Characteristics Of Included Studies

**Fig. 1:**
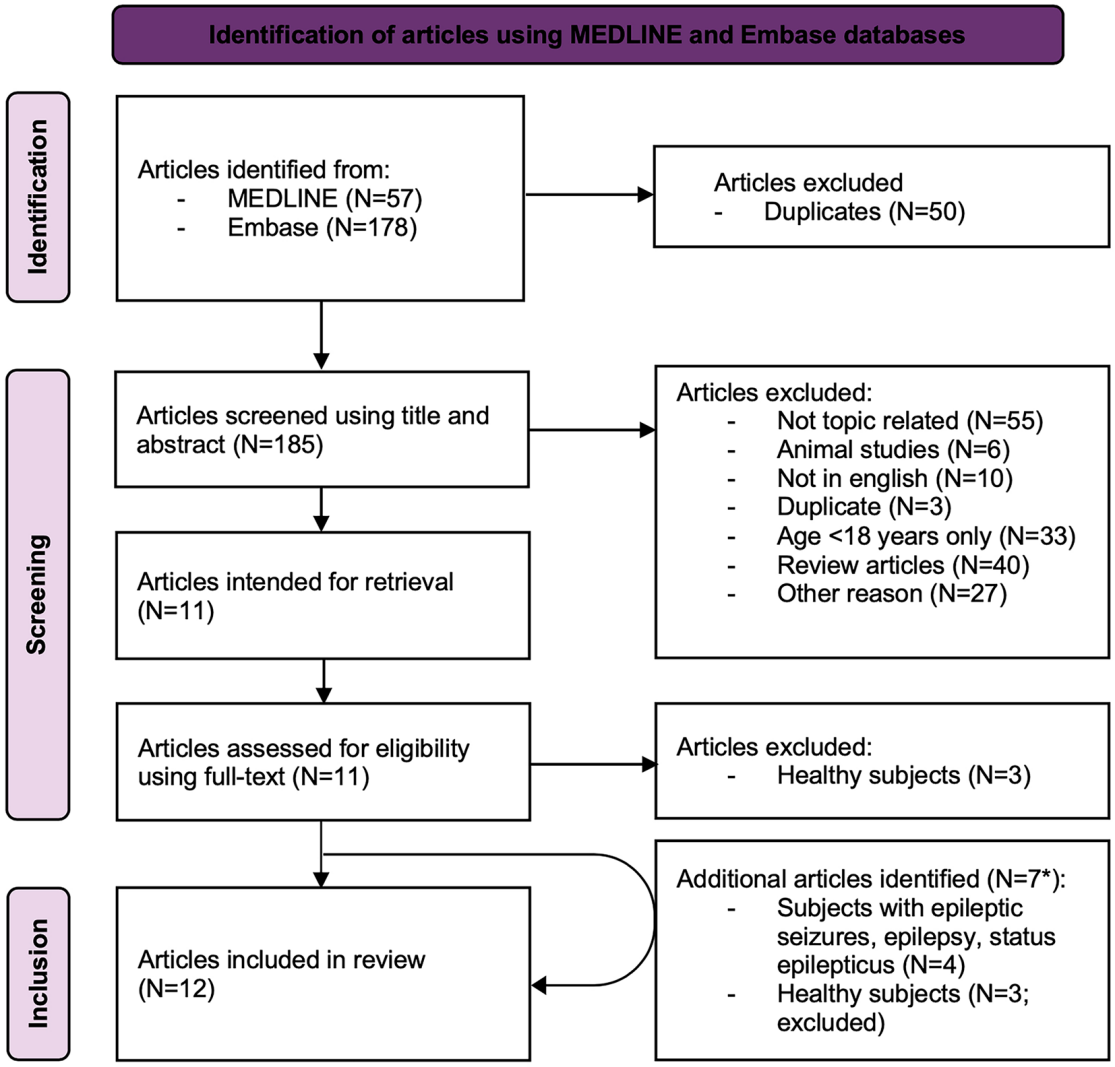
Study flow diagram. *These seven articles have been added, of which three had to be excluded because they only included healthy study participants

The screening of 185 articles identified via two databases (Embase and Medline) yielded 12 studies (including one study [17] identified during a detailed review process) with a total of 929 patients (without rigorous stratification by age group) treated with in-MDZ that addressed aspects of efficacy and tolerability of in-MDZ application for antiseizure treatment in adults (Fig. 1).

**Fig. 2:**
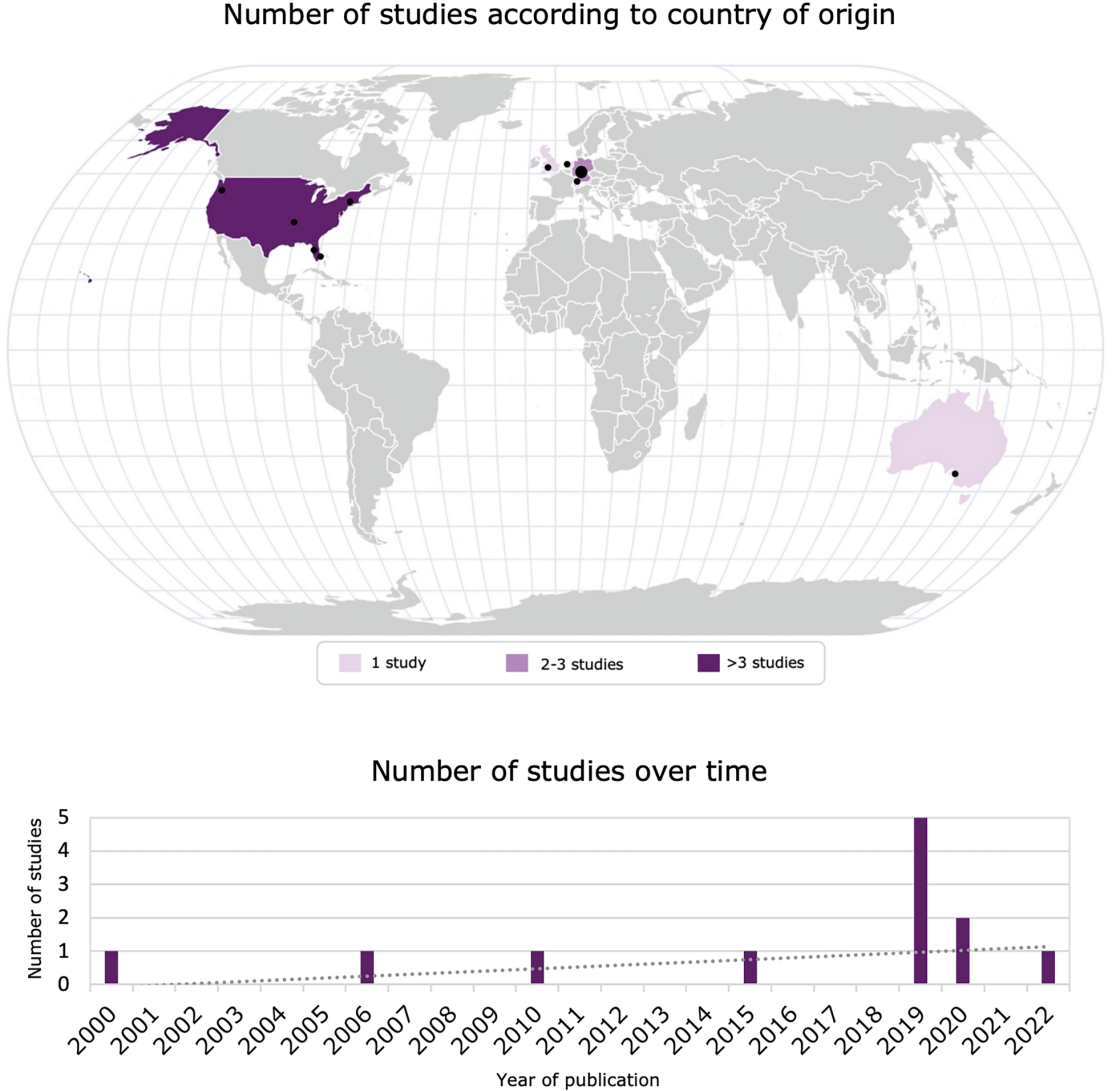
Included studies according to country of origin and over time. The geographical distribution refers to the country of the main site (as indicated by the host institution of the corresponding author). The point size corresponds to the number of studies. The Khartis software (Sciences Po Cartography Laboratory, 2017) was used to create the visualizations

Most of the included studies (66.7%) were published after 2018 [17–24]. The only two randomized trials [18, 19] were multicentric (with their main site in the United States) and accounted for 22.1% of the overall patient cohort with a total of 205 patients who received in-MDZ (Table 1). The remaining ten cohort studies, except for the study by Wheless et al. [20], were all monocentric and mostly retrospective. Among all retrospective studies, the majority were conducted in Germany, followed by other European countries (Netherlands and Switzerland), North America, and Australia (Fig. 2).

**Table 1:** Characteristics of the included studies with patients with status epilepticus, epileptic seizure, or known epilepsy

Authors		Study information									
		Publication year	Country(ies)	Number of adult patients (≥ 18 yrs) randomized to or treated with in-MDZ and setting	Condition(s)	Intervention(s)	Control/comparator(s)	Benzodiazepine pretreatment allowed	Concomittant ASD use	Age	Proportion of women
Randomized controlled studies											
Detyniecki et al^18^		2019	US, Canada, Australia, New Zealand, Germany, Hungary, Italy, Poland, Spain, Ukraine, Israel	N = 174 Setting: in‐clinic test dose phase following outpatient comparative phase)	Epileptic seizures	in-MDZ: 5 mg (first double-blind dose) 5 mg (second [optional] open-label dose)	Placebo	No	Yes	33 yrs (mean) Note: number refers to randomized population; not stratified according to patient age	51% Note: percentage refers to randomized population; not stratified according to patient age
Spencer et al^19^		2020	US, Australia, Austria, Belgium, Czechia, Germany, Lithuania, Spain	N = 31 Setting: in-clinic (epilepsy monitoring unit)	Epileptic seizures	in-MDZ (single dose): 5 mg	Placebo	Yes^c^	Yes	29 yrs (median) Note: for adult patient group	63% Note: not stratified according to patient age
Prospective cohort studies											
Wheless et al^20^		2019	US, Australia, New Zealand, Canada, Germany, Hungary, Poland, Spain, Ukraine, Israel	N = 161 patients ≥ 12 yrs Setting: outpatient Note: not stratified according to patient age	Epilepsy with history of seizure clusters^d^	in-MDZ: 5 mg (first dose) 5 mg (second dose^e^; 10 min after first dose)	None	No	Yes^f^	Not reported	Not reported
Scheepers et al^27^		2000	United Kingdom	N = 16 Setting: in-clinic	Epilepsy	in-MDZ (single dose): 5 mg (< 50 kg), 10 mg (> 50 kg)	Previously used alternative emergency medication Note: intraindividual comparison	Yes	Not reported	43 yrs (mean)	44%
Kyrkou et al^26^ (prospective?)		2006	Australia	N = 80 Setting: outpatient Note: age cutoff for adults not reported	Epilepsy (patients who had been ordered intranasal MDZ to manage prolonged seizures)	in-MDZ (not stated if single or multiple doses): 10 mg (recommended dosage)	None	Yes	Not reported	Not reported	Not reported
Retrospective cohort studies											
De Haan et al^25^		2010	Netherlands	N = 21 Setting: residential faculty of tertiary epilepsy center	Epilepsy	in-MDZ (single dose): 10 mg (each)	rec-DZP: 10 mg Note: also used as salvage therapy after in-MDZ treatment failure	No	Yes	40 yrs (mean)	38%
Kay et al^21^		2019	Germany	N = 42 Setting: in-clinic	Status epilepticus	in-MDZ (single dose): 5 mg (in 90.5%) or 2.5 mg (in 9.5%)	None	No	Yes	53 yrs (mean)	55%
Li et al^22^		2022	US	N = 38 Setting: outpatient	Epileptic seizures	in-MDZ: 5 mg (first dose) 5 mg (second dose; 10 min after first dose)	in-DZP	Yes^m^	Yes	26 yrs (median)	52%
Theusinger et al^23^		2019	Switzerland	N = 44 Setting: outpatient (emergency medical system)	Suspected epileptic seizures	in-MDZ: 0.2 mg/kg (first dose) Note: in-MDZ second dose: repetition after first dose allowed (20 mg as largest dose)	iv-MDZ: 5–15 mg im-MDZ: 10 mg DZP (rec, iv, or im): dosages not reported	Yes^o^	Patients who were saturated with ASDs in the acute phase, were excluded; no information was provided regarding ASD pretreatment	53 yrs (median)	41%
Von Blomberg et al^24^		2020	Germany	N = 224 Setting: in-clinic	Epilepsy	in-MDZ (median dose): 5 mg	No MDZ treatment Note: intraindividual comparison	No Note: only patients with prescribed in-MDZ as emergency medication	Yes (but: withdrawal of antiepileptic drugs was usually required for patients)	36 yrs (mean) Note: not stratified according to patient age	47% Note: not stratified according to patient age
Kay et al^28^		2015	Germany	N = 75 patients ≥ 12 yrs Setting: in-clinic Note: not stratified according to age	Epilepsy	in-MDZ (single dose): 5 mg (median)	No administration of in-MDZ Note: intraindividual comparison	Not reported	Yes	35 yrs (mean) Note: not stratified according to age	44% Note: not stratified according to age
Owusu et al^17^		2019	US	N = 23 Setting: in-clinic (epilepsy monitoring unit)	Epilepsy	in-MDZ (single dose) 3 mg	iv-LZP	No	Yes	40 yrs (mean)	44%

		Efficacy assessment					Safety assessment	
Authors		Clinical seizure duration (before first in-MDZ administration)	Seizure termination (after first in-MDZ/control administration)	Time to seizure termination (after first in-MDZ/control administration)	Seizure recurrence or ongoing seizure activity (in cases of more than one drug administration: first dose)		Reported side effects (proportion)	
Randomized controlled studies								
Detyniecki et al^18^		in-MDZ: 271 min (mean) Placebo: 239 min (mean) Note: numbers refer to randomized population, seizure cluster duration reported for 96% of patients; not stratified according to patient age	in-MDZ: 81%^a^ Placebo: 70% Note: percentages refer to randomized population; not stratified according to patient age	Not reported	in-MDZ (recurrence within 6 h after first administration): 41.8% Placebo: 62.7% Note: percentages refer to randomized population; not stratified according to patient age		Randomized population (overall^b^; includes double-blind [first administration] and open-label administration [if cluster did not terminate within 10 min after first dose]): in-MDZ: Nasal discomfort: 9% Somnolence: 10% Lacrimation: 1% Product taste abnormal: 3% Throat irritation: 4% Headache: 5% Placebo: Nasal discomfort: 7% Somnolence: 7% Lacrimation: 1% Product taste abnormal: 0% Throat irritation: 1% Headache: 0% Safety population: in-MDZ: Nasal discomfort: 16% Somnolence: 10% Lacrimation: 7% Product taste abnormal: 6% Throat irritation: 5% Headache: < 1% Note: not stratified according to patient age	
Spencer et al^19^		Not reported	Not reported	Not reported	Randomized population (seizure recurrence within 6 h administration): in-MDZ: 45.2% Placebo: 61.3% Note: not stratified according to patient age		in-MDZ: Nasal discomfort: 16.1% Nausea: 9.7% Throat irritation: 9.7% Product taste abnormal: 6.5% Somnolence: 6.5% Headache: 3.2% Rhinitis: 0% Suicidal ideation: 3.6% Bloodpressure: no reported differences (compared to Placebo group) that were considered clinically meaningful Placebo: Nasal discomfort: 16.1% Nausea: 3.2% Throat irritation: 9.7% Product taste abnormal: 19.4% Somnolence: 0% Headache: 9.7% Rhinitis: 6.5% Suicidal ideation: 0% Bloodpressure: no reported differences (compared to Midazolam group) that were considered clinically meaningful Note: not stratified according to patient age	
Prospective cohort studies								
Wheless et al^20^		Not reported (but: seizure clusters: episode of ≥ 2 seizures that lasted ≥ 10 min)	in-MDZ: 55%^g^ (after first dose) 80% (after second dose) Note: percentages refer to treated episodes, not patients	Not reported	in-MDZ: 31% (after first dose)		in-MDZ: Nasal discomfort: 12.4% Somnolence: 9.3% Headache: 6.2% (8.7%^h^) Fatigue: 4.3% (6.8%) Rinorrhea: 4.3% Sneezing: 3.7% Convulsion: 2.5% (5.6%) Dizziness: 2.5% (4.3%) Nausea: 2.5% (4.3%) Throat irritation: 2.5% Rhinalgia: 2.5% Product taste abnormal: 2.5% Lacrimation increased: 2.5% Note: not specified if overall or after first dose	
Scheepers et al^27^		Not reported	in-MDZ: 75%^i^ Note: two patients classified as treatment failures received buccal midazolam	Not reported	in-MDZ (recurrence within 1 h after first application): 1.2%		in-MDZ: None of the patients complained of debilitating effects on questioning	
Kyrkou et al^26^ (prospective?)		Not reported	in-MDZ: 95.4% to 96.9%^b^ (when a higher dose based on weight was administered) Note: not stratified according to patient age	Not reported	Not reported		Not systematically reported (“There were no instances of respiratory arrest, and only one report of apparent shallow breathing. Some individuals complained of discomfort or a burning sensation in the nasal passages, but this was only when they were awake for the test dose.”)	
Retrospective cohort studies								
De Haan et al^25^		Not reported	in-MDZ: 82%^j^ rec-DZP: 89%	in-MDZ: 4.3 min (mean) rec-DZP^k^: 4.6 min (mean)	in-MDZ: not reported rec-DZP: 9.5%		in-MDZ: Drowsiness: 68% Local irritation (sneezing, coughing, dry mouth, lacrimation): 29% Sedation: 8% Restlessness: 2% Headache: 0% rec-DZP: Drowsiness: 55% Local side effects: 0% Headache: 9.5% Restlessness: 9.5% Note: percentages refer to treatment episodes not cases	
Kay et al^21^		46.3 min (mean)	in-MDZ: 57%^l^ (after median dose of 5 mg)	in-MDZ: 5.1 min (mean)	in-MDZ: 35.7%		in-MDZ: Nasal irritation: 12% Prolonged sedation: 3%	
Li et al^22^		Not reported	in-MDZ^n^: 52% (after first dose) 29% (either after first or second dose) 5% (mixed response [i.e., sometimes aborting the seizures and sometimes without noticeable effect]) in-DZP: 63% (after first dose) 21% (after either first or second dose) 13% (mixed response)	Not reported	Not reported		in-MDZ: Fatigue: 24% Nasal discomfort: 10% Headache: 0% Dizziness: 0% in-DZP: Fatigue: 21% Nasal discomfort: 4% Headache: 8% Dizziness: 4% Note: not specified if overall or after first dose	
Theusinger et al^23^		Not reported	in-MDZ: 64%^p^ iv-MDZ: 57% rec-DZP: 100%	Not reported	in-MDZ: 36% iv-MDZ: 43% rec-DZP: 0%		Not reported	
Von Blomberg et al^24^		1.08 min (median)	Not reported	Not reported	in-MDZ: 50% (within 24 h after index seizure) No in-MDZ: 60% Note: not stratified according to patient age		in-MDZ: Nasal irritation: 8.1% Headache: 0.9% Cough: 2.0% Prolonged sedation: 5.7% Nausea and vomiting: 2.6% Nosebleed: 1 patient Decline in oxygen saturation (< 90%): 17% Note: number of cases; not stratified according to patient age	
Kay et al^28^		2.03 min (median) Note: not stratified according to age	Not reported	Not reported	in-MDZ: 51% (within 24 h after index seizure) No MDZ: 75% Note: not stratified according to age		in-MDZ: Nasal irritation: 4% Respiratory or circulation difficulties: 0% Note: not stratified according to age	
Owusu et al^17^		2.00 min (median)	Not reported Note: the authors report that the performance of in-MDZ and iv-LZP was comparable regarding seizure termination and prevention of status epilepticus and seizure clusters	in-MDZ: 3.2 min (median) iv-LZP: 3.3 min (median)	in-MDZ (time-to-recurrent-seizure): 13.14 h (median) Note: no percentage reported; number of repeat benzodiazepine administered within 24 h: 30.4% (in-MDZ) vs. 29.6% (iv-LZP) iv-LZP: no recurrence		in-MDZ (including seizure related): Any adverse event: 60.9% Aspiration pneumonia: 0% Transfer to intensive care unit: 0% Hypotension: 0% Respiratory depression: 0% Tongue bite: 21.7% Postictal agitation/psychosis: 0% Fatigue: 30.4% Confusion: 17.4% Shoulder dislocation: 0% Phlebitis: 0% Infiltration/extravasation: 0% iv-LZP (including seizure related): Any adverse event: 55.6% Aspiration pneumonia: 3.7% Transfer to intensive care unit: 14.8% Hypotension: 0% Respiratory depression: 0% Tongue bite: 7.4% Postictal agitation/psychosis: 14.8% Fatigue: 0% Confusion: 37.0% Shoulder dislocation: 3.7% Phlebitis: 3.7% Infiltration/extravasation: 3.7% Note: *p* = 0.002	

*ASD* antiseizure drug, *im-DZP* intramuscular diazepam, *im-MDZ* intramuscular midazolam, *in-DZP* intranasal diazepam, *in-MDZ* intranasal midazolam, *iv-DZP* intravenous diazepam, *iv-LZP* intravenous lorazepam, *iv-MDZ* intravenous midazolam, *rec-DZP* rectal diazepam

^a^Definition of successful treatment: clinical seizure termination within 10 min without recurrence 10 min to 6 h after trial drug administration

^b^Definition of successful treatment was not provided

^c^Patients with benzodiazepine pretreatment that could not be withdrawn within the washout period before the treatment phase were excluded; benzodiazepines used for rescue therapy of seizures (or for nonepileptic indications) were allowed if they were not used within 24 h prior to the trial medication administration

^d^Seizure clusters were defined as an episode of ≥ 2 seizures (focal or generalized) that lasted ≥ 10 min and had observable, stereotyped, and recognizably different pattern from patients’ noncluster seizure activity, with another seizure occurring within 6 h of cluster onset (patients with seizure clusters who experienced progression to status epilepticus were excluded)

^e^Second dose could be given if seizures did not stop within 10 min after administration of first dose or reoccurred within 10 min to 6 h

^f^Only patients on a stable antiepileptic drug regimen were included

^g^Definition of successful treatment: seizure termination ≤ 10 min after drug administration without seizure recurrence 10 min to 6 h after drug administration

^h^The first percentage refers to the period of the first 2 days after drug administration; the second percentage refers to the total percentage throughout the trial (if only one percentage is given, it applies to both periods)

^i^Definition of successful treatment: termination of the seizure

^j^Definition of successful treatment: seizure stopped within 15 min after study drug use (or a flurry of seizures was interrupted within 15 min) as well as seizure activity not recurring within 2 h after administration of the study drug

^k^Definition of successful treatment: suppressed clinical seizure activity within 15 min after study drug administration or interruption of seizure series within 15 min after study drug administration together with absence of seizure activity within 2 h

^l^Definition of successful treatment: status epilepticus stopped following the administration of intranasal midazolam (without any other drugs being given)

^m^Clobazam was used as antiseizure medication by 30.8% (diazepam group) and 28.9% (midazolam group)

^n^Definition of successful treatment: seizure stopped within 10 min after medication use

^o^A combination of midazolam and diazepam as first-line drugs was allowed (3.2% of adults were pretreated with benzodiazepine; 37% of adults first received iv-DZP)

^p^Definition of successful treatment: seizure stopped after administration of first-line drug (i.e., midazolam, diazepam; single, repeated or in combination) with no recurrence prior to arrival in the emergency department. The reported percentages refer to cases in which only the initial single drug was given (i.e., combinations excluded)

Successful treatment was mainly defined clinically as suppressed seizure activity within 10 to 15 min [18, 20, 22, 25]. The exception was two studies [17, 26] that did not explicitly define what was considered a successful treatment response (in some studies, successful treatment implied a subsequent absence of seizures for 2 to 6 h [18, 20, 25]). For seizure recurrence, the majority of studies applied a time frame of 6 h [17–20] and/or 24 h [17, 24, 27], with one study confining recurrence to a very early recurrence (within 1 h after first application) [28]. Data on seizure termination and recurrence after the first administration of in-MDZ were available for 8 of 12 studies (66.7%) (Table 1).

The in-MDZ doses administered mostly ranged from 2.5 to 10 mg per single dose [17–22, 24–28], with weight-adapted dosing reported in only one study [23] and an allowed maximum dose of 20 mg. Repetitive doses were permitted explicitly in five studies [17, 18, 20, 22, 23, 26]; in the remaining studies, repetitive administration was not permitted or specified.

Of a total of 12 included studies, two studies compared the efficacy of in-MDZ administration for seizure termination with placebo [18, 19], two studies compared the efficacy of in-MDZ administration versus no in-MDZ application within the same individuals [24, 27], and four studies compared the efficacy of in-MDZ administration with other administration routes and/or other benzodiazepines (rectal diazepam [25], intranasal diazepam [22], different regimens [intravenous midazolam, intramuscular midazolam, rectal, intravenous, or intramuscular diazepam] [23], and intravenous lorazepam [17]). One study had a comparator that was not further specified (mentioned as “previously used alternative emergency medication” [28]), and the remaining three studies [20, 21, 26] had no comparator.

### Efficacy of in-MDZ Administration for Antiseizure Treatment In Adults

**Fig. 3:**
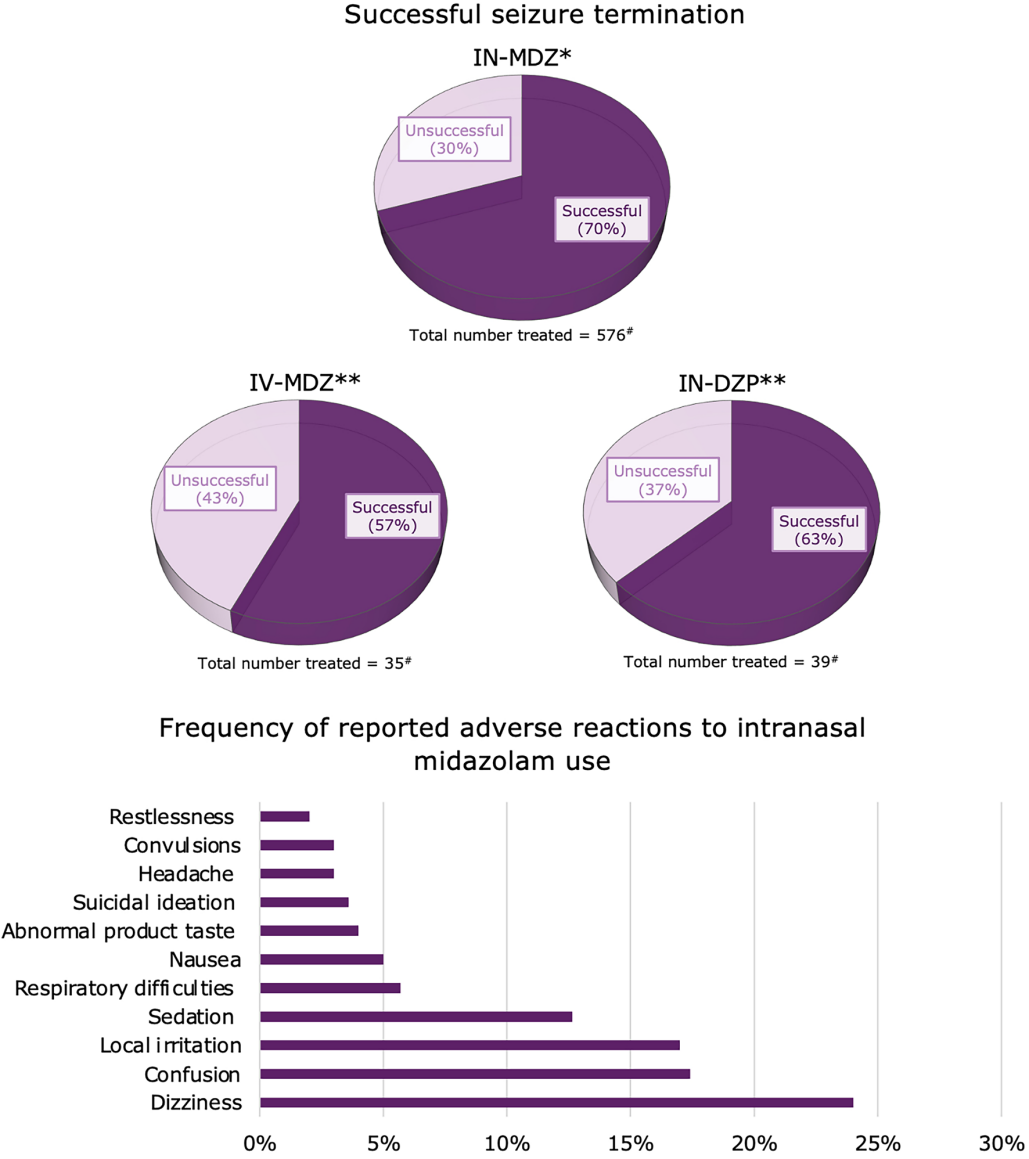
Successful seizure termination and reported side effects related to intranasal midazolam administration. *Mean value. **Percentages based only on one available study for each comparator. ^#^Total number of patients treated with available data on successful seizure termination (no stratification by age group possible due to lack of information). IN-DZP intranasal diazepam, IN-MDZ intranasal midazolam, IV-MDZ intravenous midazolam

A mean of 72.7% seizures stopped after the first administration of in-MDZ, with a standard deviation (SD) of 18% (Fig. 3). This compares to various other treatments: 70% success with placebo in one randomized study [18], 89–100% with rectal diazepam in two studies [23, 25], 63% with intranasal diazepam [22], and 57% with intravenous midazolam [23]. The highest success rate with in-MDZ was observed in studies in which a dose of 10 mg was administered or at least recommended [25, 26]. After the first in-MDZ treatment, seizures recurred in 36.5% of cases (SD 15.9%). For other treatments, recurrence rates were 61–63% with placebo [18], 60–75% without midazolam [24, 27], 0–9.5% with rectal diazepam [23, 25], and 43% with intravenous midazolam [23].

### Tolerability of Intranasal Midazolam Administration for Antiseizure Treatment in Adults

Nine of 12 (75%) studies reported side effects in detail. The most commonly reported adverse events after in-MDZ administration were dizziness (three studies [20, 22, 25]; mean 23.5% [SD 38.6%]), confusion (one study [17]; 17.4%), local irritation (eight studies [18–22, 24, 25, 27]; mean 16.6% [SD 9.6%]), sedation (eight studies [17–22, 24, 25]; mean 12.7% [SD 9.7%]), and respiratory difficulties (three studies [17, 24, 27]; mean 5.7% [SD 9.8%]; Fig. 3). Less common adverse events included nausea (three studies [19, 20, 24]; mean 4.9% [SD 4.1%]), abnormal product taste (three studies [18–20]; mean 4% [SD 2.2%]), suicidal ideation (one study [19]; 3.6%), headache (six studies [18–20, 22, 24, 25]; mean 2.6% [SD 2.7%]), convulsions (one study [20]; 3%), and restlessness (one study [25]; 2%). In the two studies with (administered or at least recommended) a higher in-MDZ dose (10 mg), there was no excess of respiratory depression reported [25, 26]. However, in one of the two studies, which reported adverse effects in detail, a relatively high proportion of patients reported drowsiness (two of three study participants) [25]. In addition, one study [17] reported that 21.7% of patients treated with in-MDZ had tongue bites compared to 7.4% in the intravenous lorazepam group (not statistically significant). Only three studies [17, 22, 25] provided data on reported side effects with either midazolam administered via different routes or other benzodiazepines.

### ROB in Included Studies

Table 2 summarizes the ROB assessment for the included studies. All included randomized trials were rated at low ROB for the randomization process, the effect of assignment to the intervention group, handling of missing outcome data, outcome measurement, and reporting of results. However, one study [18] was rated as of concern for the effect of adherence to the assigned intervention. The overall ROB was considered low because both randomized trials had a low ROB rating in most domains assessed.

**Table 2:** Risk of bias assessment

Randomized trials (RoB 2 tool)^a^																
Study	Randomization process (R)				Effect of assignment to intervention group (intention-to-treat effect [ITT])						Effect of adhering to intervention (per-protocol effect [PP])					
	R1	R2	R3	ROB	ITT1	ITT2	ITT3	ITT4	ITT5	ROB	PP1	PP2	PP3	PP4	PP5	ROB
Detyniecki et al^1^	Y	Y	N	Low	N	N	NA	NA	Y	Low	N	N	PY	PY	Y	Some concern
Spencer et al^2^	Y	Y	N	Low	N	N	NA	NA	Y	Low	N	N	N	N	Y	Low

Non-randomized trials (ROBINS-I tool)^b^																			
Study	Bias due to confounding (C)									Bias in selection of participants (S)						Bias in classification of interventions (CI)			
	C1	C2	C3	C4	C5	C6	C7	C8	ROB	S1	S2	S3	S4	S5	ROB	CI1	CI2	CI3	ROB
Wheless et al^3^	Y	NA	NA	NA	NA	NA	NA	NA	Some concern	N	NA	NA	Y	NA	Some concern	Y	Y	N	Low
Scheepers et al^4^	Y	NA	NA	NA	NA	NA	NA	NA	Some concern	N	NA	NA	Y	NA	Some concern	Y	Y	N	Low
Kyrkou et al^5^	Y	NA	NA	NA	NA	NA	NA	NA	Some concern	N	NA	NA	Y	NA	Some concern	Y	Y	N	Low
De Haan et al^6^	Y	N	NA	NA	NA	NA	NA	NA	Some concern	N	NA	NA	Y	NA	Some concern	Y	Y	N	Low
Kay et al^7^	Y	N	NA	NA	NA	NA	NA	NA	Some concern	N	NA	NA	Y	NA	Some concern	Y	Y	N	Low
Li et al^8^	Y	N	N	NA	NA	NA	NA	NA	Some concern	N	NA	NA	N	N	Some concern	Y	Y	N	Low
Theusinger et al^9^	Y	N	N	NA	NA	NA	NA	NA	Some concern	N	NA	NA	Y	NA	Some concern	Y	Y	N	Low
Von Blomberg et al^10^	Y	NA	NA	NA	NA	NA	NA	NA	Some concern	N	NA	NA	Y	NA	Some concern	Y	Y	N	Low
Kay et al^11^	Y	N	N	NA	NA	NA	NA	NA	Some concern	N	NA	NA	Y	NA	Some concern	Y	Y	N	Low
Owusu et al^12^	Y	N	N	Y	NI	N	NI	NI	Some concern	N	NA	NA	Y	NA	Some concern	Y	Y	N	Low

Randomized trials (RoB 2 tool)^a^																
Study	Missing outcome data (M)					Outcome measurement (O)						Reported results (RR)			Overall risk of bias (ROB)	
	M1	M2	M3	M4	ROB	O1	O2	O3	O4	O5	ROB	RR1	RR2	ROB	Overall ROB	Overall predicted direction of bias
Detyniecki et al^1^	Y	NA	NA	NA	Low	N	PN	N	NA	NA	Low	PY	N	Low	Low	NA
Spencer et al^2^	Y	NA	NA	NA	Low	N	N	N	NA	NA	Low	NI	N	Low	Low	NA

Non-randomized trials (ROBINS-I tool)^b^																								
Study	Bias due to deviations from intended interventions (DI)							Bias due to missing data (M)						Bias in measurement of outcomes (MO)					Bias in selection of reported results (SR)				Overall risk of bias (ROB)	
	DI1	DI2	DI3	DI4	DI5	DI6	ROB	M1	M2	M3	M4	M5	ROB	MO1	MO2	MO3	MO4	ROB	SR1	SR2	SR3	ROB	Overall ROB	Overall predicted direction of bias
Wheless et al^3^	PN	NA	NA	PY	N	NA	Some concern	Y	N	NA	NA	NA	Low	Y	Y	NA	PN	High	N	N	N	Low	Some concern	NA
Scheepers et al^4^	PN	NA	Y	Y	Y	NA	Low	Y	N	NA	NA	NA	Low	Y	Y	NA	PN	High	N	N	N	Low	Some concern	NA
Kyrkou et al^5^	PN	NA	NA	NI	NI	NA	High	Y	N	NA	NA	NA	Low	Y	Y	NA	PN	High	N	N	N	Low	Some concern	NA
De Haan et al^6^	PN	NA	NI	PY	PY	NA	Some concern	Y	N	NA	NA	NA	Low	PN	PN	Y	PN	Low	N	N	N	Low	Low	NA
Kay et al^7^	PN	NA	NA	PY	NI	NA	Some concern	Y	N	NA	NA	NA	Low	N	N	Y	PN	Low	N	N	N	Low	Some concern	NA
Li et al^8^	PN	NA	PY	PY	PY	NA	Low	Y	N	NA	NA	NA	Low	PY	Y	Y	PN	High	N	N	N	Low	Some concern	NA
Theusinger et al^9^	PN	NA	N	PY	Y	N	Some concern	N	N	NA	NI	NI	Some concern	PY	Y	Y	PN	High	N	N	N	Low	Some concern	NA
Von Blomberg et al^10^	PN	NA	Y	PY	Y	NA	Low	PY	N	NA	NA	NA	Low	PY	Y	NA	PN	High	N	N	N	Low	Some concern	NA
Kay et al^11^	PN	NA	Y	PY	PY	NA	Low	PY	N	NA	NA	NA	Low	PY	Y	Y	PN	High	N	N	N	Low	Some concern	NA
Owusu et al^12^	PN	NA	Y	Y	Y	NA	Low	Y	N	N	NA	NA	Low	PY	Y	Y	PN	High	N	N	N	Low	Some concern	NA

*N* no, *NA* not applicable, *NI* no information, *PN* probably no, *PY* probably yes, *ROB* risk of bias, *Y* yes

^a^R1: Random allocation sequence? R2: Allocation sequence concealed? R3: Do baseline differences (between intervention groups) suggest a problem with the randomization process? ITT1: Were participants aware of their assigned intervention group? ITT2: Were carers/people delivering the interventions aware of participants’ intervention group assignment? ITT3: If Y/PY to ITT1 or ITT2: Were these deviations likely to have affected the outcome? ITT4: If Y/PY to ITT3: Were these deviations from intended intervention balanced between groups? ITT5: Was an appropriate analysis used to estimate the effect of intervention group assignment? PP1: Were participants aware of their assigned intervention group? PP2: Were carers/people delivering the interventions aware of participants’ intervention group assignment? PP3: Were there failures in the implementation of the intervention that could have affected the outcomes? PP4: Was there non-adherence to the assigned intervention regimen that could have affected the outcomes? PP5: Was an appropriate analysis used to estimate the effect of adhering to the intervention? M1: Were data for this outcome available for all (or nearly all) randomized individuals? M2: If N/PN/NI to M1: Is there evidence that the results was not biased by missing outcome data? M3: If N/PN to M2: Could missingness in the outcome depend on its true value? M4: If Y/PY/NI to M3: Is it likely that missingness in the outcome depended on its true value? O1: Was the method for outcome measurement inappropriate? O2: Could measurement (or ascertainment) of the outcome have differed between intervention groups? O3: If N/PN/N information to O1 and O2: Were outcome assessors aware of the intervention allocation? O4: If Y/PY/NI to O3: Could assessment of the outcome have been influenced by knowledge of received intervention? O5: If Y/PY/NI to O4: Is it likely that outcome assessment was influenced by knowledge of received intervention? RR1: Were the data that produced this result analyzed in accordance with a pre-specified analysis plan that was finalized before unblended outcome data were available for analysis? RR2: Is the numerical result being assessed likely to have been selected on the basis of the results from multiple eligible outcome measurements (e.g. scales, definitions, time points) within the outcome domain?

^b^C1: Is there potential for confounding of the intervention effect (if N/PN to C1: the study can be considered to be at low risk of bias due to confounding and no further signaling questions need to be considered)? C2: If Y/PY to C1: Was the analysis based on splitting participants follow-up time to intervention received (if N/PN: see question relating to baseline confounding [C4]; if Y/PY: see question C3)? C3: Were intervention discontinuations (or switches) likely to be related to factors that are prognostic for the outcome (if N/PN: see questions relating to baseline confounding [C4]; if Y/PY: see questions relating to both baseline and time-varying confounding [C6 and C7])? C4: Baseline confounding only: Did the authors use an appropriate analysis method that controlled for all important confounders? C5: If Y/PY to C4: Were confounders that were controlled for measured validly and reliably by the variables available? C6: Baseline confounding only: Did the authors control for any post-intervention variables that could have been affected by the intervention? C7: Baseline and time-varying confounding: Did the authors use an appropriate analysis method that controlled for all important confounders including time-varying confounding? C8: If Y/PY to C7: Were confounders that were controlled for measured validly and reliably by the variables available? S1: Was selection of participants based on participant characteristics observed after the start of intervention? S2: If Y/PY to S1: Were the post-intervention variables that influences selection likely to be associated with intervention? S3: If Y/PY to S2: Were the post-intervention variables that influenced selection likely to be influenced by the outcome or a cause of the outcome? S4: Do start of follow-up and start of intervention coincide for most participants? S5: If Y/PY to S2 and S3, or N/PN to S4: Were adjustment techniques used to correct for the presence of selection bias? CI1: Were intervention groups clearly defined? CI2: Was the information used to define intervention groups recorded at the start of the intervention? CI3: Could classification of intervention status have been affected by knowledge of the outcome (or risk of the outcome)? DI1: Assignment to intervention: Were there deviations from the intended intervention (beyond what would be expected in usual practice)? DI2: Assignment to intervention: If Y/PY to DI1: Were these deviations unbalanced between groups and likely to have affected the outcome? DI3: Starting and adhering to intervention: Were important co-interventions balanced across intervention groups? DI4: Starting and adhering to intervention: Was the intervention implemented successfully for most participants? DI5: Did participants adhere to the assigned intervention regimen? DI6: If N/PN to DI3, DI4 or DI5: Did the authors use an appropriate analysis method to estimate the effect of starting and adhering to the intervention? M1: Were outcome data available for all (or nearly all) participants? M2: Were participants excluded due to missing data on intervention status? M3: Were participants excluded due to missing data on other variables needed for the analysis? M4: If PN/N to M1, or Y/PY to M2 or M3: Are the proportion of participants and reasons for missing data similar across interventions? M5: If PN/N to M1, or Y/PY to M2 or M3: Is there evidence that results were robust to the presence of missing data? MO1: Could the outcome measure have been influenced by knowledge of the intervention received? MO2: Were outcome assessors aware of the intervention allocation? MO3: Were the methods of outcome assessment comparable across intervention groups? MO4: Were any systematic errors in measurement of the outcome related to intervention received? SR1: Is the reported effect estimate likely to be selected on the basis of the results from multiple outcome measurements within the outcome domain? SR2: Is the reported effect estimate likely to be selected on the basis of the results from multiple analyses of the intervention-outcome relationship? SR3: Is the reported effect estimate likely to be selected on the basis of the results from different subgroups?

The included nonrandomized studies [17, 20–28] were all rated as of some concern for risk of confounding and selection bias. The ROB in classifications of interventions was considered low in all studies. In contrast, the ROB for deviations from the planned interventions was rated as low in only five of nine studies. For the remaining studies, there was at least some concern about the ROB in this domain. All but one study (the latter was rated as of concern) were classified as low ROB because of missing data. Eight studies were rated as having a high ROB regarding the measurement of outcomes. All studies were considered low risk in the domain of bias risk in the selection of reported results. Overall, the ROB was graded as moderate for the nonrandomized studies because most studies raised at least some concern about the overall ROB.

## Discussion

Rapid and easy administration of benzodiazepines can be challenging in the acute care setting in patients with prolonged epileptic seizures, seizure clusters, or status epilepticus. From an application perspective, in-MDZ administration appears to be particularly promising. This route of administration seems less invasive compared to intravenous, intramuscular, or intraosseous routes and less stigmatizing compared to rectal administration and is thus of potential clinical relevance, particularly regarding an administration by a layperson. To date, to the best of our knowledge, no systematic review has examined the existing global evidence on the efficacy and tolerability of in-MDZ administration for antiseizure treatment in adults.

Within the framework of this systematic review, we have compiled the available evidence from the international literature, including 12 identified studies published within the last 38 years, with most published over the previous 5 years. Most of the included studies provided data regarding the proportion of successful seizure termination and the recurrence of seizures after the first in-MDZ administration. The intranasal administration led to a mean seizure termination in nearly three of four patients. Compared with the reported successful seizure termination rates with other benzodiazepines or midazolam administered via alternative routes (except for rectal midazolam), in-MDZ administration did not appear to be inferior to other benzodiazepine alternatives (i.e., intravenously administered midazolam, diazepam given via intranasal route). It should be noted that the few studies in which comparatively higher doses (10 mg per single dose) were administered or at least recommended had the highest rates of successful seizure termination, which might suggest a dose–response relationship. The reported higher rates of successful seizure termination (and lower recurrence rates) under rectal diazepam might be attributable, in part, to the longer half-life of diazepam [29]. This could be relevant because the definition of successful seizure termination was linked to seizure absence (over a variable period) immediately following the first dose in some studies. The high rate of spontaneous seizure termination in the placebo group is in line with evidence from the literature that most epileptic seizures are self-limiting and last less than a few minutes [1]. However, the evidence for the efficacy of in-MDZ is overall low because only five studies (of which only one was a randomized controlled trial) compared in-MDZ administration to a comparator (Supplemental Table 1). From a pharmacokinetic perspective, it should be noted that rectal administration might not be as efficient compared to most other routes of administration because of slower absorption rates through the mucosa compared to all other routes of administration [4]. In addition, rectal emergency treatment is not universally accepted and is associated with adverse psychosocial effects, such as embarrassment, increased stigmatization, social fear, and inconvenience in administration [30]. Further points that should be considered when assessing efficacy in terms of seizure termination is that first-line doses of administrated in-MDZ varied by up to fourfold across the included studies, and successful seizure termination was not defined uniformly across the individual studies. Considerable evidence suggests that underdosing of benzodiazepines is one of the main reasons for the failure of seizure termination in the context of status epilepticus and should therefore be considered when interpreting these results [31–33]. Furthermore, there is a relatively wide range of seizure termination rates, ranging from 52% to almost 97%. This observed variation could be partially explained by the inhomogeneous cohorts and the lack of standardization regarding reported seizure termination and recurrence rates. Given that most studies were not randomized, this may be relevant because baseline characteristics that may have influenced the response to benzodiazepine acute therapy (such as underlying seizure etiology and subsequent different concomitant antiepileptic treatments) may have been unequally distributed. These concerns are reinforced by the ROB assessment of the nonrandomized studies, which attests to some concerns with most of the included studies, especially regarding the outcome assessments.

Regarding reported side effects, in-MDZ administration appeared safe and associated mainly with only moderate accompanying symptoms, such as dizziness, sedation, and local irritation. The latter seems specific to the intranasal route of administration but may not be necessarily midazolam-related, although comparisons with other intranasally administered benzodiazepines were scarce (with only one study investigating intranasal diazepam administration [22]). This is supported by the latest results of the randomized IN-MIDAZ study (published after the screening period) [14]. The study investigated the efficacy and safety of in-MDZ and intramuscularly administered midazolam for the termination of epileptic seizures (clinical and electroencephalographic) in 130 study participants (both pediatric and adult patients). Local side effects were seen more frequently in the intranasal group, with hypotension occurring more commonly as a severe adverse effect in the intramuscular group. In terms of efficacy, there was a significant advantage for intramuscular application, although the mean clinical seizure termination time was still less than 2 min (53.9 ± 25.8 s vs. 104.3 ± 66.4 s; *p* = 0.002). The authors concluded that in-MDZ represents a useful option for seizure interruption.

The extent to which a dose dependence (i.e., 5- vs. 10-mg in-MDZ single dose) of the reported side effects may exist cannot be conclusively assessed. There is weak evidence that higher doses may be associated with increased side effects, such as drowsiness [25]. However, the study suggesting this did not report excess respiratory depression [25]. Of the three studies that recorded respiratory difficulties, one showed a decline in oxygen saturation (< 90%; without necessary intubation in any patient) [24], whereas the other two reported no recorded respiratory difficulties [17, 27]. It should be noted that these three studies were retrospective and thus without a placebo control. Respiratory failure is not always related to the use of benzodiazepines in seizing patients, as respiratory depression and failure have been reported as leading complications (80%) in patients with convulsive status epilepticus [34] and are independent predictors of death in this context [35]. Dizziness and sedation have been described previously in association with other midazolam administration routes and are also described for other benzodiazepines [4, 36, 37]. Yet in the studies included in this systematic review, there were no reports of adverse effects under alternative benzodiazepine treatments in most cases.

Limitations include the following:

1. That most included studies were nonrandomized. This results in an inherent risk of systematic bias, especially for retrospective designs (e.g., recall bias in retrospective questioning about side effects).
2. The high heterogeneity of the studies considered for this systematic review. This applies, for instance, to the age of the patients included in the study. The age cutoff (pediatric versus adult) was not always clearly stated, and outcomes were not always stratified by age group.
3. The different definitions of efficacy outcomes used.
4. The largely missing blinding for therapy allocation is relevant in the context of adverse event reporting, which possibly introduced a bias in the reported frequency of adverse events.

Strengths of this review are the following:

1. The consideration of control or comparative interventions consisting of the administration of other benzodiazepines or midazolam with other administration routes, which places the efficacy and safety assessment of in-MDZ in the context of other commonly used acute therapies and thus contributes to better comparability.
2. That this review, despite the aforementioned heterogeneity of the included studies, provides the most comprehensive overview of the available evidence on the efficacy and tolerability of in-MDZ for antiseizure treatment in adults to date. Given the limited data available on this topic, we consider the investigation of in-MDZ application in the context of either epilepsy, epileptic seizure(s), or status epilepticus to be justified despite differences in prognosis.

## Conclusions

In summary, this systematic review provides evidence for the safe and effective use of in-MDZ against epileptic seizures in adults. However, a high ROB concerning outcome measurement was noted in 8 of 12 included studies, which needs to be considered when interpreting the efficacy of in-MDZ compared to other benzodiazepines or routes of administration. In this regard, this study provides supportive evidence for current clinical practice and calls for further prospective studies with larger sample sizes and a lower ROB.

## Supplementary Information

Below is the link to the electronic supplementary material.

Supplementary file1 (DOCX 37 kb)

## Data Availability

Data are available from the corresponding authors upon reasonable request.

## Clinical Trial Registration

CRD42022369040 (PROSPERO study ID).
