# Supplementary material for: Efficacy and Tolerability of Intranasal Midazolam Administration for Antiseizure Treatment in Adults: A Systematic Review
Source: Neurocrit Care. 2024 Apr 5;41(2):632–50. doi: 10.1007/s12028-024-01971-x (PMC11377482; doi:10.1007/s12028-024-01971-x)
Supplement: Supplementary file 1 — Supplementary file1 (DOCX 37 kb) [file 12028_2024_1971_MOESM1_ESM.docx]

**Supplemental Material**

EFFICACY AND TOLERABILITY OF INTRANASAL MIDAZOLAM ADMINISTRATION FOR ANTISEIZURE TREATMENT IN ADULTS: A SYSTEMATIC REVIEW

Tolga D Dittrich, MD*; Dominik Vock, MD*; Urs Fisch, MD; Lisa Hert, MD; Sira Baumann, MD; Paulina Kliem, MD; Stephan Rüegg, MD; Stephan Marsch, MD; Gian Marco De Marchis, MD; Raoul Sutter, MD

* equal contribution

**Table of contents**

[Supplemental text section 1: Detailed description of the search strategy 2](#_Toc130455432)

[Supplemental Table 1. Grading of recommendations assessment, development and evaluation 2](#_Toc130455433)

# **Supplemental text section 1: Detailed description of the search strategy**

The search algorithm used was: midazolam.mp. [mp=ti, ab, hw, tn, ot, dm, mf, dv, kf, fx, dq, bt, nm, ox, px, rx, an, ui, sy], limit 1 to yr="1985 - 2022", nasal.mp. [mp=ti, ab, hw, tn, ot, dm, mf, dv, kf, fx, dq, bt, nm, ox, px, rx, an, ui, sy], spray.mp. [mp=ti, ab, hw, tn, ot, dm, mf, dv, kf, fx, dq, bt, nm, ox, px, rx, an, ui, sy], seizure.mp. [mp=ti, ab, hw, tn, ot, dm, mf, dv, kf, fx, dq, bt, nm, ox, px, rx, an, ui, sy], status epilepticus.mp. [mp=ti, ab, hw, tn, ot, dm, mf, dv, kf, fx, dq, bt, nm, ox, px, rx, an, ui, sy], epilepsy.mp. [mp=ti, ab, hw, tn, ot, dm, mf, dv, kf, fx, dq, bt, nm, ox, px, rx, an, ui, sy], 2 and 3, 2 and 4, 8 or 9, 5 or 6 or 7, 10 and 11, remove duplicates from 12.

# **Supplemental Table 1. Grading of recommendations assessment, development and evaluation**

| **Certainty assessment** | | | | | | | **№ of patients** | | **Effect** | | **Certainty** | **Importance** |
| --- | --- | --- | --- | --- | --- | --- | --- | --- | --- | --- | --- | --- |
| **№ of studies** | **Study design** | **Risk of bias** | **Inconsistency** | **Indirectness** | **Imprecision** | **Other considerations** | **Intranasal midazolam** | **Other benzodiazepine(s) or midazolam with other administration route or placebo** | **Relative**  **(95% CI)** | **Absolute**  **(95% CI)** |  |  |
| **Seizure termination** | | | | | | | | | | | | |
| 4 | randomized controlled and observational | serious | serious | not serious | not serious | dose-response relationship with partially different initial doses administered | 229/300 (76.3%) | 154/211 (73.0%) | **RR 1.05** (0.80 to 1.37) | **36 more per 1.000** (from 146 fewer to 270 more) | ⨁⨁◯◯  LOW | CRITICAL |

**CI:** confidence interval; **RR:** risk ratio
